# Egg-White Proteins Have a Minor Impact on the Bactericidal Action of Egg White Toward *Salmonella* Enteritidis at 45°C

**DOI:** 10.3389/fmicb.2020.584986

**Published:** 2020-10-08

**Authors:** Florence Baron, Marie-Françoise Cochet, Mariah Alabdeh, Catherine Guérin-Dubiard, Michel Gautier, Françoise Nau, Simon C. Andrews, Sylvie Bonnassie, Sophie Jan

**Affiliations:** ^1^STLO, INRAE, Institut Agro, Rennes, France; ^2^School of Biological Sciences, University of Reading, Reading, United Kingdom; ^3^UFR Sciences de la Vie et de l’Environnement, Université de Rennes I, Rennes, France

**Keywords:** *Salmonella* enteritidis, egg white, egg-white proteins, bactericidal, membrane potential, *psp* response

## Abstract

*Salmonella enterica* serovar Enteritidis is noted for its ability to survive the harsh antibacterial activity of egg white which is presumed to explain its occurrence as the major food-borne pathogen associated with the consumption of eggs and egg products. Liquid egg white is a major ingredient for the food industry but, because of its thermal fragility, pasteurization is performed at the modest temperature of 57°C (for 2–6 min). Unfortunately, such treatment does not lead to sufficient reduction in *S.* Enteritidis contamination, which is a clear health concern when the product is consumed without cooking. However, egg white is able to limit *S.* Enteritidis growth due to its alkaline pH, iron deficiency and multiple antimicrobial proteins. This anti-*Salmonella* activity of egg white is temperature dependent and becomes bactericidal once the incubation temperature exceeds 42°C. This property is exploited in the highly promising pasteurization treatment (42–45°C for 1–5 days) which achieves complete killing of *S.* Enteritidis. However, the precise mechanism and the role of the egg-white proteins are not fully understood. Here, the impact of exposure of *S.* Enteritidis to egg white-based media, with or without egg-white proteins (>10 kDa), under bactericidal conditions (45°C) was explored by measuring survival and global expression. Surprisingly, the bactericidal activity of egg white at 45°C was only slightly affected by egg-white proteins indicating that they play a minor role in the bactericidal activity observed. Moreover, egg-white proteins had minimal impact on the global-gene-expression response to egg white such that very similar, major regulatory responses (20% genes affected) were observed both with and without egg-white proteins following incubation for 45 min at 45°C. Egg-white proteins caused a significant change in expression for just 64 genes, including the *psp* and lysozyme-inhibitor responses genes which is suggestive of an early membrane perturbation effect. Such damage was supported by disruption of the proton motive force by egg-white proteins. In summary, the results suggest that low-mass components of egg white are largely responsible for the bactericidal activity of egg white at 45°C.

## Introduction

Egg white provides a nutritional reserve for the developing embryo and also affords protection against invasive microorganisms; this is achieved through a broad spectrum of antimicrobial factors. Egg white contains an arsenal of antimicrobial molecules, including: lysozyme, which lyses Gram-positive bacteria*;* ovotransferrin, which deprives bacteria of iron; protease inhibitors, which inhibit exogenous proteases; vitamin-binding proteins that impose vitamin deficiency; and minor proteins and peptides with predicted antibacterial effects (Baron et al., 2016 for review). Previous studies on the antimicrobial activity of chicken egg white have largely focused on *Salmonella enterica* serovar Enteritidis as this serotype is the major food-borne pathogen (90%) related to the consumption of eggs and egg products (EFSA Biohaz Panel (European Food Safety Autority Panel on Biological Hazards), 2014). The high occurrence of *S.* Enteritidis in egg-related salmonellosis is due to its specialized ability to survive egg-white exposure (Lock and Board, 1992; Clavijo et al., 2006; Guan et al., 2006; Gantois et al., 2008b, 2009).

Microbiological status is a critical factor for table eggs and egg products because liquid whole egg, egg yolk and egg white are used in the manufacture of various foodstuffs (sausages, sauces, cakes, pasta, dessert, etc.) and it is clearly important that such egg products are safe, especially when processing does not include a cooking step. Industrial liquid egg products are pasteurized but the traditional heat treatment of liquid egg white (e.g., 57°C for 2–6 min) does not provide high-level killing of *S.* Enteritidis, although it does preserve the desirable organoleptic/quality aspects of the egg white (Baron, 2010).

Egg white exhibits strong anti-bacterial properties, but this effect is temperature dependent. For *S*. Enteritidis, weak growth is observed in egg white at 20 and 30°C, but a bactericidal action is observed at 42°C (Baron et al., 2016 for review). It is notable that the temperatures where bactericidal activity of egg white is observed are similar to those naturally encountered during egg formation (i.e., close to hen’s body temperature, 42°C); 42°C is routinely used in studies on the bactericidal activity of egg white (Guan et al., 2006; De Vylder et al., 2013; Raspoet et al., 2014). The impact of temperature on the antimicrobial activity of egg white is underlined by a patent describing a novel egg-white pasteurization method whereby liquid egg white is incubated at 42–45°C for 1–5 days resulting in stability of the egg white at room temperature for several months (Liot and Anza, 1996). This method provides a complete killing of *S.* Enteritidis and is superior (in terms of reduction in bacterial numbers) to the less efficient, but more traditional, “egg-white pasteurization” treatment (57°C for 2–6 min) that requires subsequent storage under refrigeration (Baron, 2010).

To better understand egg-white antibacterial mechanisms, we previously examined the global gene expression response of *S.* Enteritidis to egg-white model medium (EWMM) exposure for 45 min at 45°C (Baron et al., 2017). This study confirmed that, after 24 h incubation, *S.* Enteritidis suffers bacteriostasis upon exposure to egg white at moderate temperature (30°C) but is lysed at 45°C (although not in Tryptone Soy Broth, TSB, optimal medium). Further, exposure to EWMM at 45°C for 45 min caused a major change in global gene expression indicative of a substantial modification of *S.* Enteritidis physiology. Such changes included induction of responses to nutrient deprivation (iron and biotin) and cell damage/stress (envelope stress, heat shock, translation inhibition, alkaline stress), as well as a shift in energy metabolism and catabolism (reduced respiration, TCA cycle activity, motility and taxis, and raised glycolysis and hexonate/hexuronate catabolism). The egg-white induced expression effects observed reflect the attempts of *S.* Enteritidis to overcome the antibacterial activities of egg white and support the view that *S.* Enteritidis suffers damage during egg-white exposure that causes the cell death observed after 24 h of incubation at 45°C (Baron et al., 2017).

The present study focuses on the role that the egg-white proteins play in the killing activity of egg white at 45°C. Among the EW proteins known to have an antimicrobial effect, lysozyme (14.3 kDa), ovotransferrin (77.7 kDa), protease inhibitors (12–780 kDa), and lipocalins (Ex-FABP; 21 kDa) are considered to be the major contributors (Baron et al., 2016 for review). Since these proteins have a molecular weight higher than 10 kDa, ultrafiltration using a 10 kDa cut-off membrane provides an EW fraction depleted of the major EW proteins. Thus, we compared the survival of *S.* Enteritidis at 30 and 45°C, and in response to a range of egg-white protein levels using the following media: egg white, EW; egg-white filtrate, EWF (EW free of proteins, i.e., free of molecules > 10 kDa); and egg-white model medium, EWMM (EWF with 10% EW added). We also employed microarray analysis to investigate the effect of egg-white proteins on global expression of *S.* Enteritidis at 45°C using EWF as the comparator. The results suggest that EW proteins play only a minor role in the bactericidal activity of EW toward *S.* Enteritidis at 45°C and have a limited impact on the gene expression response.

## Materials and Methods

### Bacterial Strain

*Salmonella* Enteritidis NCTC13349 was kindly donated by Matthew McCusker (Center for Food Safety and Food Borne Zoonomics, Veterinary Sciences Centre, University College Dublin, Ireland). This strain was isolated from an outbreak of human food poisoning in the United Kingdom that was traced back to a poultry farm. The stock cultures were stored at −80°C in 50% (v/v) glycerol. Before use, cells were propagated twice overnight at 37°C in Tryptone Soy Broth (TSB, Merck, Darmstadt, Germany) without shaking.

### Preparation of Egg White-Based Media

Egg white (EW) was prepared from 5–10 day-old eggs obtained from a conventional hen housing system. Eggshell surfaces were cleaned with tissue paper, checked for cracks and then surface sanitized with 70% alcohol. Residual alcohol was removed by briefly flaming the shell. Eggshells were then broken, under sterile conditions, and the released egg whites were combined and aseptically homogenized with a DI25 Basic homogenizer (Ika, Grosseron, Saint-Herblain, France) at 9,500 rpm for 1 min. The EW pH was 9.3 ± 0.1. Egg white filtrate (EWF) was prepared by ultrafiltration of EW. Ultrafiltration was performed using a pilot unit (TIA, Bollène, France) equipped with an Osmonics membrane (5.57 m^2^, 10 kDa cut-off; PW 2520F, Lenntech B.V., Delft, Netherlands). Filtration was achieved according to Baron et al. (1997). Concentrated EW (retentate) was circulated back to the feedtank and permeate (filtrate) was drained off, collected in a beaker, sterilized by filtration (Nalgene^®^ filter unit, pore size <0.2 μm, Osi, Elancourt, France), and then stored at 4°C until use. The EWF pH was 9.3 ± 0.1.

Egg white model medium (EWMM) was prepared by adding 10% EW (vol/vol) to EWF. The solution was then homogenized with a DI25 basic homogenizer at 9,500 rpm for 1 min. The EWMM pH was 9.3 ± 0.1. EWMM and EWF were analyzed by a 12% SDS-PAGE stained with 0.2% Coomassie blue to check protein contents: no bands were visible for EWF, confirming the absence of EW proteins (>10 KDa) in this medium in comparison with EWMM (10% of EW). Media sterility was routinely checked by inoculating Tryptone Soy Agar (TSA, Merck, Darmstadt, Germany) plates with 1 mL of medium and then confirming lack of colony formation after overnight incubation at 37°C.

### Exposure of *S.* Enteritidis to Egg White-Based Media

After propagation in TSB, bacterial suspensions were centrifugated at 5,600 × g at 15°C for 7 min, and cells were washed three times with EWF. The final pellet was resuspended in the original volume of EWF and was then inoculated at a final concentration of 6.9 ± 0.4 log_10_ CFU/mL into EW, EWMM or EWF. The inoculated media were incubated at 30 or 45°C for 45 min and 24 h to evaluate their bactericidal activities. Bacterial suspensions of *S.* Enteritidis (overnight cultures centrifuged at 5,600 × g and 15°C for 7 min, and washed three times with fresh optimum medium, TSB) were also incubated at a final concentration of 6.9 ± 0.4 log_10_ CFU/mL in TSB at pH 7.3 for 24 h at 30 and 45°C, as a control. Three experiments were performed for each condition.

For survival curves, bacterial suspensions of *S.* Enteritidis were prepared as described above and incubated for 24 h either in TSB, EW, EWMM or in EWF. Samples were collected for enumeration at different time intervals, from 0 to 24 h. Experiments were repeated three times.

For transcriptome analysis by microarray and qRT-PCR, bacterial suspensions of *S.* Enteritidis were prepared and incubated in EWMM or in EWF at 45°C as previously described. RNA extraction was carried out in samples collected at the time of inoculation (0 min) and after 45 min of incubation (45 min).

### Enumeration of Bacterial Cells After Incubation

An enumeration method based on miniaturization of the conventional plate-counting technique was used according to Baron et al. (2006) with a TSA overlay procedure. After incubation at 37°C for 20–24 h, the number of colony forming units (CFU) was recorded. When necessary, results were compared using analysis of variance and the average comparison test using the R 2.13.0 software^1^.

### Membrane Depolarization

Membrane depolarization measurements were performed using a method adapted from Epand et al. (2010) and based on the use of DiSC_3_(5), a lipophilic potentiometric dye exhibiting fluorescence intensity changes in response to alterations in transmembrane potential. Bacterial cells from an overnight culture in TSB at 37°C were diluted 100 times in TSB and incubated for 3.5 h at 37°C. Bacteria in mid-log phase were centrifuged (5,600 g for 7 min at 15°C), washed three times in a buffer (5 mM Hepes with 5 mM glucose, pH 7.2) and finally diluted to an OD_600 *nm*_ of 0.6 in the same buffer. Then a stock solution of DiSC_3_(5) was added to a final concentration of 1 μM. KCl was also added to the cell suspension to a final concentration of 100 mM to equilivance the cytoplasmic and external K^+^ concentrations. After allowing the dye signal to stabilize in the dark for 15 min at 37°C, the cells charged with the dye were diluted 10 times in each medium (5 mM Hepes, Hepes 5 mM with 15 μg/mL Mellitin, EW, EWMM, EWF) and incubated at 30 or 45°C. After 10 min of incubation, changes in fluorescence caused by the disruption of the membrane potential gradient (ΔΨ) across the cytoplasmic membrane were recorded with a spectrofluorometer (Molecular Devices Spectra, MAX Gemini XS, San José, EU) at an excitation wavelength of 622 nm and an emission wavelength of 670 nm. Results expressed in Relative Fluorescence Unit (RFU) were compared using analysis of variance and the average comparison test using the R 2.13.0 software (see text footnote 1).

### RNA Isolation

After incubation, cells were centrifuged at 10,000 g at 4°C for 5 min. The pellets were immediately frozen in liquid nitrogen and stored at −80°C. After thawing on ice, cells were disrupted by treatment with TE (10 mM Tris-HCl pH 7, 1 mM EDTA) buffer, containing 20 mg/mL lysozyme, for 30 min at 37°C. Then, cells were mechanically lysed with zirconium beads using a FastPrep-24 instrument (MP Biomedicals, Illkirch, France); two 30 s cycles at 30 Hz were applied, interspaced with 30 s cooling periods. Total RNA was then isolated by phenol-chloroform extraction. RNA quantity and quality were assessed spectrophotometrically by measuring the UV absorbance profile (NanoDrop, NanoDrop Technologies, Inc., Rockland, Wilmington, DE, United States) at 230, 260, and 280 nm. For the microarray experiment, additional analysis for RNA integrity was performed using an RNA 6000 Nano LabChip kit (2100 Bioanalyzer, Agilent Technologies, Santa Clara, CA, United States). The RNA samples were then DNase-treated using a DNA-free kit (Ambion, Austin, TX, United States) according to the manufacturer’s instructions. Quantification of the RNA and any contamination by proteins was again assessed using a NanoDrop ND-1000 and RNA integrity again confirmed using a 2100 Bioanalyzer. The resultant total RNA (500 ng of each sample) was reverse transcribed and labeled using the SuperScriptTM Indirect cDNA Labeling System (Invitrogen, Life Technologies, Courtaboeuf, France) according to the protocol provided by the manufacturer, except that the hexamer solution was replaced with the pdN6 hexamer solution (Roche Diagnostics, Meylan, France).

### Microarray and Experimental Design

The DNA microarray was designed using the published genome sequence for *S.* Enteritidis strain NCTC13349^2^. The microarray, consisting of probes matching 3971 ORFs (representing 94.4% of *S.* Enteritidis gene composition), was designed using Agilent’s e-array software^3^. Note that 235 ORFs were not included due to restricted design parameters of the e-array program. The custom oligonucleotide microarray was manufactured by Agilent Technologies using an 8 × 15 K format and included each probe in duplicate. Microarray hybridization and data analysis were performed by the Pasteur Institute (Transcriptome and Epigenome Platform PF2, Paris, France).

*S.* Enteritidis exposed for 45 min to EWF at pH 9.3 was compared to a reference (0 min incubation, i.e., *S.* Enteritidis washed with EWF at pH 9.3). For each time point, three arrays were hybridized with three independent biological replicates in duplicate (giving three biological replicates and two technical replicates). Cy3 and Cy5 dye-swap design was included in order to reduce dye-specific effects. The data have been deposited in NCBI’s Gene Expression Omnibus and are accessible through GEO Series accession number GSE144179^4^.

To compare the genes differentially expressed between EWF and EWMM, the data obtained from a previous study for exposure of *S.* Enteritidis to EWMM (at pH 9.3 for 45 min) were used (Baron et al., 2017)^5^.

### Data Acquisition and Pre-processing of Microarray Data

Images of the microarrays were scanned using an Axon 4000a scanner (Axon, Instruments, CA, United States) and intensity data were extracted using the GenepixPro 6.1 software. Raw microarray data were first normalized using the LOWESS (Locally Weighted Scatter Plot Smoother) regression option in the R software suite, version 2.10.1^6^, to correct for dye-bias within the array, followed by median normalization to normalize across all arrays.

### Differential Expression Analysis of Microarray Data

Changes in gene expression upon incubation were recorded as fold changes (ratio at 45 min cf. 0 min, in EWF; ratio in EWF cf. EWMM, at 45 min). Statistical significance was estimated with a moderated *t-*test using the LIMMA package (Smyth, 2004) of the R software suite (version 2.10.1). Genes that exhibited change in expression (with respect to the control) with *P* ≤ 0.05 were considered as differentially regulated. Differentially expressed genes were initially categorized by function according to the Clusters of Orthologous Groups^7^ designations and categorization was subsequently optimized manually.

### Confirmation of Selected Genes by qRT-PCR Analysis

Confirmation of the transcriptomic analysis was achieved using qRT-PCR. Primers (Table 1) for amplification of selected genes were designed using Primer 3^8^. Non-contamination of RNA by gDNA was confirmed by qPCR prior to cDNA synthesis. cDNA was synthesized using the high-capacity cDNA Reverse Transcription Kit (Applied Biosystems) as recommended by the manufacturer. qRT-PCR was performed using an iCycler iQ Real-Time PCR Detection System (Bio-Rad). Thermal cycling consisted of 5 min at 95°C, followed by 45 cycles of 15 s at 95°C, 20 s at 60°C and 40 s at 72°C. A melting curve analysis (55–95°C) was performed after the thermal profile to ensure specificity and PCR efficiency was calculated at between 85 and 105% from the log-linear portion of the standard curves. RNA extractions were performed on *S*. Enteritidis incubated in EWMM or EWF at 45°C for 0 and 45 min. RNA extracts thus obtained was subjected to qRT-PCR, in triplicate, for each selected gene. Standard curves were generated to calculate the copy number of each gene in each sample. The three most stable control genes under the conditions used here were determined by geNorm^9^ from five potential genes. qRT-PCR data were normalized by geometric averaging of three internal control genes (*asmA*, *emrA*, *orf32*; primers in Table 1). Changes in gene expression upon incubation were recorded as fold change (ratio at 45 min cf. 0 min, in EWF; ratio in EWF cf. EWMM, at 45 min). To highlight the differential gene expression in the two media, the ratio between the fold change at 45 min in EWMM and the fold change in EWF was calculated for each selected gene.

**TABLE 1:** Sequences of primers used for qPCR.

**Gene**	**Forward primer**	**Reverse primer**
*asmA*	ACCGGACACGTTCAGGTAAC	GGCAACAGGTTGTCCAGATT
*emrA*	ATCTGTGGGTGGACGCTAAC	CCATATCCAGACCGACGACT
*orf32*	CGGCTCTTTAACGCTCTGAC	CCGGTGGGTTTTGATAAATG
*pspG*	TGTTACCGTGGCTACTGCTG	GAGGCAAACCGTTTTCTTGA
*argA*	GGGCTGCGTAAATTGTTTGT	ATATCGACAGGCGTGAAACC
*pnuC*	AGATGCTGGGGTTACAGGTG	GCGTGTCATCAAAATCATCG
*cysD*	GCTGCCACTTCACGGATAAT	CAAAAACGACTCACCCACCT
*ybdA*	TGCTCAATCTCAGCCTGTTG	GTGGAGTGCGTCATCATTTG

## Results

### Egg-White Proteins Have a Minor Impact on the Rapid Killing of *S.* Enteritidis by Egg White at 45°C

In order to better understand the impact of EW proteins on killing of *Salmonella*, *S.* Enteritidis was subjected to EW, EW filtrate (EWF; EW free of proteins > 10 kDa) and EW model medium (EWMM; EWF supplemented with 10% EW) at pH 9.3 (the pH of EW a few days after laying), and at either 30 or 45°C. For comparison, the effect of *S*. Enteritidis exposure to TSB (rich growth medium, pH 7.3) was also examined. Each medium was inoculated with 6.9 ± 0.2 log_10_ CFU/mL. Over the relatively short 45 min incubation time employed, no significant change in viable cell number occurred (7.1 ± 0.2 log_10_ CFU/mL) under any of the conditions tested.

**FIGURE 1:**
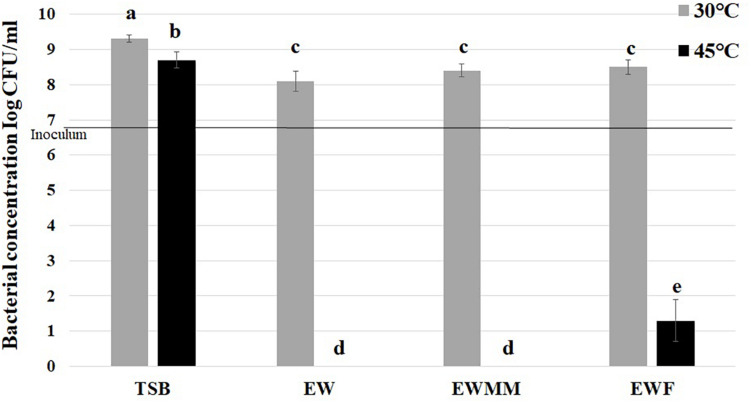
*S.* Enteritidis survival after 24 h at 30 or 45°C in TSB (control), EW, EWF (free of EW proteins of >10 kDa) and EWMM (EWF with 10% EW). Inoculum levels were 6.9 ± 0.2 log_10_ CFU/mL. Means of triplicate experiments are shown with standard deviations. For each condition, results were compared using analysis of variance and the average comparison test using R software. Letters identify averages that are significantly different (*p* ≤ 0.05).

After 24 h exposure at 30°C, *S.* Enteritidis exhibited modest growth in EW, EWMM and EWF (increases of 1.2, 1.5, and 1.6 log_10_ CFU/mL, respectively) which was significantly weaker (*p* = 0.05) than that obtained in TSB medium (increases of 2.4 log_10_ CFU/mL) (Figure 1). These observations confirm that EW and EW-based media impose a bacteriostatic influence on *S.* Enteritidis at 30°C. In contrast, during 24 h incubation at 45°C, a bactericidal effect was observed for EW, EWMM and EWF whereas weak growth (increase of 1.8 log_10_ CFU/mL) was obtained in TSB (Figure 1). These results demonstrate the importance of temperature for the *Salmonella*-killing activity of EW-based media.

**FIGURE 2:**
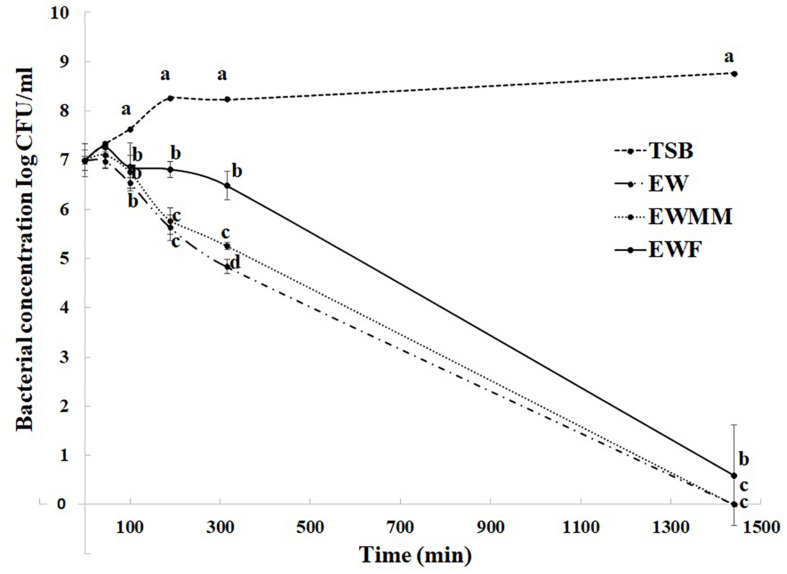
Survival curves of *S.* Enteritidis in EW media at 45°C. Media employed were TSB (control), EW, EWF (free of EW proteins of > 10 kDa), and EWMM (EWF with 10% EW). Means of triplicate experiments are shown with standard deviations. For each time, results between media were compared using analysis of variance and the average comparison test using the R software. Letters were used to identify averages that are significantly different (*p* ≤ 0.05).

The survival curves at 45°C (Figure 2) are similar for EW and EWMM with no detectable bacteria (<0 CFU/mL) after 24 h of incubation. This similarity validates the use of EWMM as a model medium of EW for the transcriptional study since it allows easy recovery of *S.* Enteritidis RNA due to its lower viscosity with respect to EW. Surprisingly, the survival curve obtained in EWF also showed a drastic reduction of viable cell number, although this was slightly (but significantly) lower than that observed in EWMM and EW. From 190 min, the number of survivors differs by 1 log_10_ CFU/mL for *S.* Enteritidis exposed to EWF compared to *S.* Enteritidis exposed to EW or EWMM, and over the 24 h incubation period the reduction observed was of 5.9 log_10_ CFU/mL for EWF and 6.9 log_10_ CFU/mL for EW and EWMM, respectively. This result suggests, somewhat surprisingly, a relatively minor role for EW proteins (>10 kDa) in killing of *S.* Enteritidis in EW at 45°C.

### Effect of Egg-White Proteins on *S.* Enteritidis Gene Expression at 45°C

To further explore the impact of EW proteins on the bactericidal effect of EW at 45°C, global gene expression changes caused by incubation in EWF were investigated by microarray analysis. For this purpose, an early incubation time point (45 min) was selected corresponding to the killing phase during which *S.* Enteritidis would still be largely viable and present at levels enabling recovery of sufficient RNA for expression analysis. In contrast to 24 h incubation, 45 min of exposure resulted in no significant decline in cell number (Figure 2), indicating that the culture was in the early phase of EWMM-induced cell death and/or damage.

**FIGURE 3:**
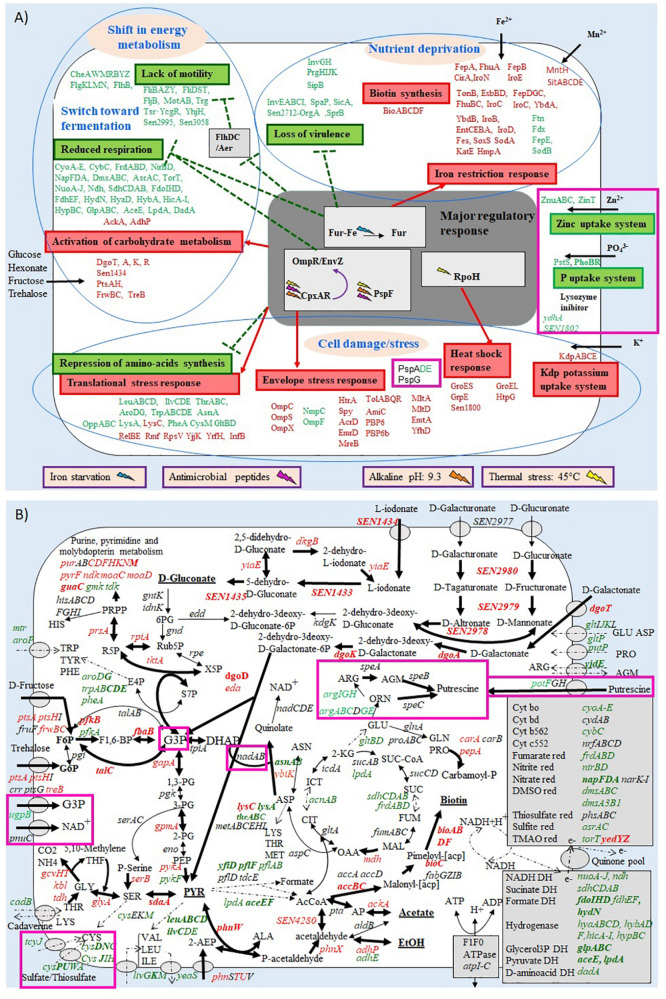
Summary of the effect of *S.* Enteritidis 45 min exposure to EWF **(A,B)** on selected metabolic pathways. Transporters are symbolized by gray ovals. Up-regulated, down-regulated, and non-regulated genes are represented in red, green or black, respectively. Genes with a ≥4-fold change in expression are in bold. The metabolites predicted to be increased are underlined. Up-regulated, down-regulated and non-regulated pathways are represented by thick, broken, or fine arrows, respectively. Genes with different expression levels between the incubation in EWMM (from Baron et al., 2017) and in EWF are boxed in pink. Metabolic pathway data were obtained from KEGG (www.genome.jp/kegg).

Gene expression of *S.* Enteritidis after 45 min exposure to EWF was compared to a reference (0 min incubation) and genes with a statistically significant change in expression (*p* ≤ 0.05) were considered as differentially regulated. Thus, after 45 min incubation in EWF, 23.25% of genes were differentially (>2-fold) regulated (380 induced and 598 repressed). A high proportion of the genome was thus subject to expression alteration in EWF, similar to the proportion reported in our previous study (18.7%) after 45 min incubation in EWMM (Baron et al., 2017). Moreover, the gene expression response to EWF exposure at 45°C revealed the same major effects observed upon exposure to EWMM at 45°C (Baron et al., 2017): (i) a micronutrient deprivation response (induction of biotin biosynthesis genes, induction of iron-uptake genes and repression of iron-rationing genes); (ii) a shift in energy metabolism and catabolism (reduced respiration, TCA cycle activity, motility and taxis, and raised glycolysis and hexonate/hexuronate catabolism); and (iii) a cell damage/stress response (envelope stress, heat shock, translation inhibition, induction of the Kdp potassium uptake system) (Figure 3).

In order to identify those genes that are differentially expressed in response to the presence/absence of EW proteins, microarray data obtained from our previous study (*S.* Enteritidis exposure to EWMM for 45 min at 45°C; Baron et al., 2017) was compared to that obtained with EWF at 45 min. A statistical comparison between the fold changes obtained for each gene after 45 min exposure to either EWMM or to EWF at 45°C showed that only 64 genes exhibited a significant difference of greater than 2-fold (Table 2).

**TABLE 2:** Genes of *S.* Enteritidis differentially expressed in EWMM (with EW proteins) and in EWF (free of EW proteins of > 10 kDa).

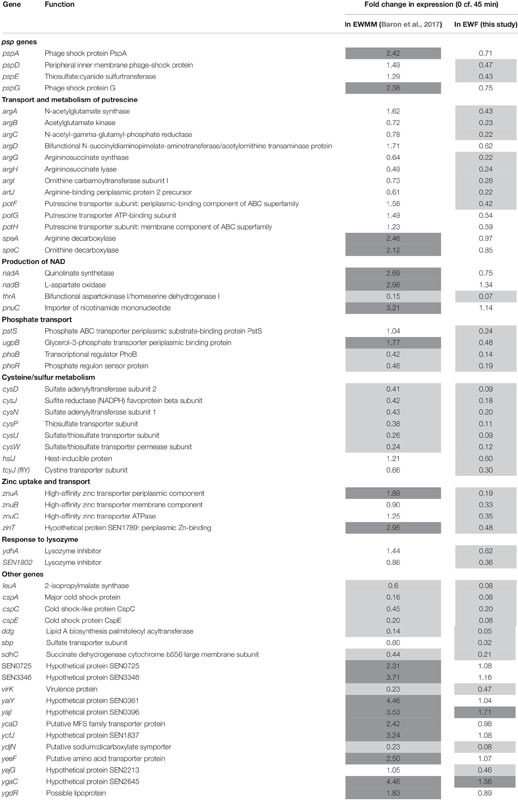

**Highlighted expression changes correspond to significantly (p*≤ *0.05) up-regulated (dark gray) or down-regulated (light gray) changes after 45 min exposure to EWMM or EWF. Only genes exhibiting a ≥2-fold expression difference ratio between EWMM (data from our previous study Baron et al., 2017) and EWF (data from this study) after 45 min exposure at 45°C were listed in this table.**

#### Confirmation of Microarray Data by qRT-PCR

To confirm the validity of the expression effects observed by microarray analysis, qRT-PCR was used to determine the expression changes of five genes of relevance following incubation in EWMM or EWF for 45 min at 45°C (Table 3). The genes selected for analysis belong to the principal functional groups observed in Table 2. The qRT-PCR data confirm the expression change of the five selected genes (*pspG*, *argA*, *pnuC*, *cysD, ydhA*) of *S.* Enteritidis in response to EWMM in comparison to EWF, although the qRT-PCR data indicates a greater degree of expression change than seen by microarray analysis, an effect observed previously when comparing microarray and qRT-PCR results (Franchini, 2006). The ratio between the fold change of the two media are comparable (mean of 3.32-fold and of 4.88 for microarray and qRT-PCR data, respectively). Thus, the qRT-PCR data provide support for the reliability of the expression effects revealed by microarray analysis (Table 2).

**TABLE 3:** Confirmation of selected genes by qRT-PCR analysis.

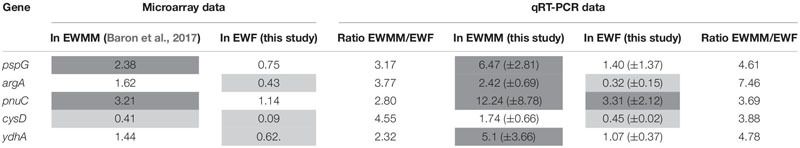

**Microarray data are included to allow direct comparison with the qRT-PCR data. Calculation of fold change by qRT-PCR was determined from the number of RNA copies after 45 min of incubation divided by the number of RNA copies at 0 min of incubation. Data were normalized using values for three internal control genes (asmA, emrA, orf32). Standard deviations data are in parentheses. Highlighted fold changes correspond to significantly (p = 0.05) up-regulated (dark gray) and down-regulated (light gray) genes. The ratio between the fold change at 45 min in EWMM and EWF was calculated from microarray data (from Baron et al., 2017 for EWMM and this study for EWF) and from qRT-PCR analysis (this study) for each selected gene.**

#### *psp* Genes Induction

**FIGURE 4:**
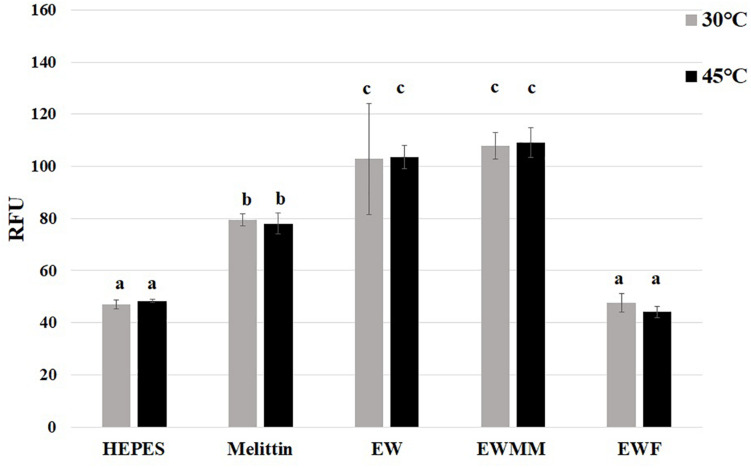
Effect of EW proteins on the membrane depolarization of *S.* Enteritidis. *S*. Enteritidis cells were charged with diSC_3_(5) dye and incubated at 30 or 45°C in Hepes (negative control, 5 mM pH 7.2), melittin (positive control, 15 μg/mL in 5 mM Hepes, pH 7.2) and EW, EWMM, or EWF at pH 9.3. Results are shown as means of relative fluorescence units (RFU) of triplicate experiments with standard deviations. Results obtained under the conditions indicated were compared using analysis of variance and the average comparison test using the R software. Letters were used to identify average significantly different (*p* ≤ 0.05).

The microarray data (Table 2) show that the *pspADE* and *pspG* genes were induced in EWMM (1.29–2.42-fold-change depending on the gene), but repressed in EWF (0.43–0.75-fold-change depending on the gene) after 45 min at 45°C. These results suggest that EW proteins are responsible for the induction of these genes since EWMM and EWF have the same pH (9.3), were incubated at the same temperature (45°C), and only differ in protein content (EW proteins present in EWMM, absent in EWF). The *psp* genes encode “phage-shock proteins” that are induced in response to various membrane-altering stresses and dissipation of the proton motive force (pmf) (Darwin, 2005; Jovanovic et al., 2006). To further explore any pmf disruption effect of EW proteins, the membrane depolarization of *S*. Enteritidis was compared in EWMM, EWF and EW at 30 and 45°C (Figure 4) using the diSC3(5) fluorescent dye method (Epand et al., 2010).

The results show that the relative fluorescence intensity was not affected by temperature (30 vs. 45°C) under any of the conditions tested. However, there was a significant increase in diSC3(5) fluorescence for *S*. Enteritidis incubated in the presence of EW proteins (in EW or EWMM) whereas no such increase was observed in the absence of EW proteins (in EWF). In EWF the relative fluorescence intensity remained similar to that of the negative control (Hepes buffer) suggesting that EWF does not cause pmf disruption under the conditions tested. The relative fluorescence intensity measured in EW and EWMM were similarly high and ∼20% greater than that measured for the positive control (Hepes buffer with mellitin). These results thus indicate that EW proteins are required for the disruption of *S.* Enteritidis membrane potential mediated by EW in the early stage of exposure at 45°C.

#### Transport and Metabolism of Putrescine

A significant differential expression in EWMM vs. EWF was observed for the *argA, argCBH, argD*, and *argI* genes involved in the production of ornithine and arginine from glutamate, and for *artJ* that encodes a periplasmic binding protein of an L-arginine ABC transporter (Table 2). The *speA* and *speC* genes involved in the biosynthesis of putrescine from arginine or ornithine, respectively, also showed a significant difference in expression in EWMM vs. EWF. In addition, the *potFGH* genes that encode an ATP-dependent putrescine transporter were differentially expressed in the two media. All of these genes involved in putrescine production (Schneider and Wendisch, 2011) exhibited higher relative expression in their presence EW proteins (i.e., in EWMM) than in its absence (i.e., in EWF). This differential expression effect indicates that EW proteins induce an increase in putrescine accumulation in *S.* Enteritidis incubated for 45 min at 45°C.

#### Production of NAD

The *nadAB* and *pnuC* genes also showed a differential expression response to EW proteins with a significantly greater relative expression in EWMM with respect to EWF (Table 2). The products of these genes are involved in the biosynthesis of NAD. NAD is an essential cofactor in numerous biological reactions and organisms have developed different ways of producing it through both “*de novo*” and salvage pathways (Rodionov et al., 2008). The products of the *nadAB* genes are involved in the formation of quinolinic acid from L-aspartate and dihydroxyacetone phosphate (DHAP). These reactions lead to *de novo* synthesis of NAD. The product of the *pnuC* gene is involved in a salvage pathway utilizing exogenous nicotinamide mononucleotide as a source of pyridine. As the derepression of these genes is related to the need for enhanced NAD biosynthesis when internal NAD levels are low, the observed induction by EW proteins suggests a greater *S.* Enteritidis demand for NAD following exposure for 45 min at 45°C to EWMM than to EWF.

#### Phosphate Transport

The *pstS* gene is strongly down-regulated in EWF but not in EWMM (Table 2). This gene belongs to the *pstSCAB* cluster that encodes a phosphate-specific ABC transporter complex. The *pstSCAB* cluster is under control of the PhoBR regulon (Marzan and Shimizu, 2011). Interestingly, the expression of *phoBR*, encoding the PhoBR two-component transcriptional regulator of the phosphate regulon, was also more strongly down-regulated in EWF than in EWMM. Moreover, the *ugpB* gene, which is also under control of PhoBR, showed a significant differential expression in the two media. The *ugpB* gene specifies the periplasmic binding protein of the UgpBAECQ ATP-binding cassette transporter responsible for the acquisition of glycerol-3-phosphate (G3P). This latter gene was down-regulated in EWF (0.48-fold-change after 45 min) and slightly, but significantly, induced in EWMM (1.77-fold-change after 45 min). These results suggest that EW proteins raise expression of genes belonging to PhoBR regulon in these conditions (exposure to 45°C for 45 min).

#### Cysteine/Sulfur Metabolism

A group of genes belonging to the *cys* regulon also showed differential expression between EWF and EWMM (Table 2). The three *cys* operons involved in sulfate assimilation and cysteine biosynthesis (*cysDNC*, *cysJIH*, *cysPUWAM*) were more strongly repressed in EWF in comparison with EWMM. The *tcyJ* gene (formerly *fliY*) that encodes a periplasmic binding protein of a cystine/cysteine ABC transport system was also more greatly repressed in EWF in comparison to EWMM. The gene *hslJ*, also belonging to the Cys regulon (Lilic et al., 2003) and encoding an outer membrane lipoprotein, was significantly repressed in EWF and slightly (but not significantly) induced in EWMM. Thus, EW proteins caused an apparent increase in cysteine biosynthesis and uptake capacity in the early stage of exposure to 45°C.

#### Zinc Uptake and Transport

A further difference in expression between EWMM and EWF was observed for the *znuABC* genes encoding the ZnuABC transporter and *SEN1789* encoding ZinT, an accessory component contributing to zinc recruitment. These zinc-uptake genes were strongly down-regulated in EWF (from 0.19 to 0.48-fold-change after 45 min incubation at 45°C) but exhibited a raised expression response in EWMM (from 0.90 to 2.35 relative expression after 45 min incubation at 45°C) (Table 2). As the expression of *znuABC* and *zinT* is linked to zinc homeostasis (Gabbianelli et al., 2011), their induction in the presence of EW proteins suggests a reduced level of zinc availability when EW proteins are present.

#### Response to Lysozyme

The *ydhA* (or *mliC*) and the *SEN1802* genes encode proteins which inhibit the activity of lysozyme (Deckers et al., 2004). These genes were significantly down-regulated in EWF but showed greater relative expression in EWMM after 45 min at 45°C (Table 2). This indicates that these genes are subject to induction by the presence of EW proteins, which include lysozyme. Thus, this may represent a direct response to the lysozyme component of EW.

Nineteen other genes showing a differential expression response between the two media are also listed in Table 2, many of which are hypothetical in nature. A notable observation is that the major effects are the same in both media for 45 min exposure at 45°C (summary in Figure 3). The differential expression effect of EW proteins observed here is relatively modest both in terms of the number of genes (2% of the total genome) significantly affected (highlighted in Figure 3), and the degree of expression change achieved (average difference of 3-fold by microarray).

## Discussion

### Egg-White Proteins <10 kDa Do Not Have a Major Role in the Bactericidal Activity of EW at 45°C

After 24 h incubation at 30°C, *S.* Enteritidis grew weakly in EW, EWMM and EWF whereas a bactericidal effect was observed at 45°C. At 20–30°C, all previous studies report weak growth in EW, from 1 to 2 log_10_ CFU/mL, depending on the strain and incubation time (Clay and Board, 1991; Lock and Board, 1992; Humphrey and Whitehead, 1993; Růzicková, 1994; Schoeni et al., 1995; Baron et al., 1997; Cogan et al., 2001; Chen et al., 2005; Murase et al., 2005). However, once the temperature is raised to ∼42°C, previous studies report that EW exhibits a bactericidal effect from <2 to 3.5 log_10_ CFU/mL after 24–96 h incubation (Guan et al., 2006; Kang et al., 2006; Alabdeh et al., 2011). A strong bactericidal activity of EW at 45°C (reduction of ∼7 log_10_ CFU/mL) was also reported by Jan et al. (2013) for *E. coli* and *S.* Enteritidis (Baron et al., 2017). The temperature studied here (45°C) is higher than that of the hen reproductive tract (42°C), but it matches the recommendations of the patent of Liot and Anza (1996) which describes an original process for liquid EW pasteurization based on incubation at moderate temperatures (42–45°C) for a prolonged duration (1–5 days). Thus, 45°C was chosen in the present study to investigate the response of *S.* Enteritidis to EW pasteurization at 45°C and to reveal the underlying bactericidal mechanisms. The understanding of these mechanisms could be relevant for the food industry in that they could suggest possibilities for further optimization of the control of *S*. Enteritidis in egg products. The results clearly show that bactericidal action requires the combined action of temperature (45°C) with EW (or EW-based media) as *S.* Enteritidis is not subject to substantial killing in TSB at 45°C nor in EW, EWMM, EWF at 30°C.

The results also show that EW and EWMM exhibit a complete bactericidal effect against *S.* Enteritidis after 24 h of incubation at 45°C and confirm that EWMM can be used for research purposes in place of EW. EWMM (composed of EWF with 10% EW) was previously shown to be an appropriate model of EW to study *S.* Enteritidis behavior (Baron et al., 1997; Alabdeh et al., 2011; Baron et al., 2017). Since the major EW proteins known to have an antimicrobial effect have a molecular weight higher than 10 kDa, ultrafiltration using a 10 kDa cut-off membrane provides an EW fraction free of the major EW proteins, here designated “EW filtrate” (EWF). Hence, EWF can be viewed as the aqueous phase of EW, with the same pH value of 9.3 but depleted of EW proteins (Baron et al., 1997), and can be used to specifically investigate the role of EW proteins (<10 kDa) on the bactericidal activity observed at 45°C. Thus, in the present study, the comparison of the effect of incubation of *S.* Enteritidis in EWF and EWMM (EWF supplemented with 10% EW) allows any impact of EW proteins (greater than 10 kDa) on *S*. Enteritidis survival to be separated from the effect of the EWF.

The complete loss of *S.* Enteritidis viability after 24 h incubation in EWF at 45°C, and the very similar results obtained with EW and EWMM (Figures 1, 2), indicate that EW proteins <10 kDa play a relatively minor role in the bactericidal effect observed at 45°C.

### The Global Transcriptional Responses of *S.* Enteritidis to EWF and EWMM Exposure for 45 min at 45°C Are Very Similar

Surprisingly, the transcriptional response of *S*. Enteritidis to 45 min incubation in EWF at 45°C was very similar to that observed previously during incubation in EWMM (Baron et al., 2017). In both cases, there were major responses to: nutrient deprivation (iron and biotin); cell damage/stress; and a shift in energy metabolism and catabolism (Figure 3).

#### Response to Nutrient Deprivation

The *bio* operon, involved in biotin synthesis and subject to induction under biotin limitation, was up-regulated in EWF (Figure 3) to the same degree as observed in EWMM (Baron et al., 2017). This operon was also up-regulated in *S.* Enteritidis exposed to 80% EW at 37°C (Huang et al., 2019) and a quantitative proteomic analysis also showed that biotin-synthesis-related proteins are induced by EW (Qin et al., 2019). Further, a role for the *bioB* gene has been suggested in EW survival at 42°C (Raspoet et al., 2014) and Wang et al. (2018) showed a reduced survival ability in EW for a *bioC* knock-out mutant. This is consistent with biotin limitation due to its chelation by avidin present in EW (Banks et al., 1986). The avidin-biotin complex (69 kDa) is the strongest known non-covalent interaction (K_*d*_ = 10^–15^ M) between a protein and ligand (Banks et al., 1986) and is expected to be retained by the ultrafiltration membrane and this would explain why the *bio* operon is induced both in EWF and EWMM.

The same iron-restriction responses were observed in EWF (Figure 3) and EWMM (Baron et al., 2017) with induction of iron-uptake genes, repression of iron-rationing genes and the same regulation of genes controlled by Fur, the regulator of the iron-uptake machinery in response to iron availability (Andrews et al., 2003; Bjarnason et al., 2003; McHugh et al., 2003). These results corroborate numerous studies suggesting the major antimicrobial role of iron deficiency in EW (Garibaldi, 1970; Lock and Board, 1992; Baron et al., 1997) and highlight the role of Fur in mediating the response of *S*. Enteritidis to poor iron availability (Kang et al., 2006; Baron et al., 2017; Huang et al., 2019; Qin et al., 2019). It is considered that essentially all the iron present in EW is bound to ovotransferrin (Baron et al., 2016) and this protein (77.7 kDa) would have been lost from the EWF along with any associated iron such that EWF would be expected to impose an iron deficiency similar to that of EWMM. A similar down-regulation of virulence related genes, all members of the Fur modulon, was also observed in the two media (Figure 3).

#### Shift in Energy Metabolism and Catabolism

Again, the gene expression profiles in EWF and EWMM (Baron et al., 2017) showed very similar changes in energy-generation systems; this may represent an adaptation to the conditions encountered in these media. The changes involved a shutdown of flagella and motility gene expression, and a loss of respiratory and TCA cycle gene expression (Figure 3). There was increased carbohydrate uptake and glycolysis gene expression along with raised expression of genes involved in serine breakdown via the One-Carbon pathway. The combined up-regulation of glycolysis and down regulation of both the TCA cycle and respiration suggest a shift in energy metabolism from respiration to fermentation. The genes encoding mixed-acid fermentation enzymes (*ackA, adhP*) were also induced in both media suggesting the accumulation of ethanol and acetate.

#### Cell Damage and Envelope Stress

Exposure to EWF and EWMM led to similar degrees of expression change for genes involved in amino-acid synthesis, the translational stress response, heat shock stress, and the Kdp potassium uptake system (Figure 3). Similarly, genes involved in the cell-envelop stress response also showed similar expression changes in EWF and EWMM (Figure 3). Such genes included the *spy* gene encoding a periplasmic chaperone protein, the *htrA* (*degP*) gene encoding a periplasmic/membrane-associated serine endoprotease that degrades abnormal proteins, the genes specifying the Tol-Pal system involved in the maintenance of cell-envelope integrity, the *omp* genes encoding outer-membrane porins, and genes encoding peptidoglycan hydrolases and multidrug-efflux systems that remove antimicrobial compounds. Changes in porin-expression profile, remodeling of peptidoglycan mediated by peptidoglycan hydrolases, activation of multidrug efflux pumps and degradation of abnormal protein are recognized as antimicrobial resistance mechanisms (Delcour, 2009; Price and Raivio, 2009). All these genes are under control of the CpxAR regulator that responds to a variety of envelope perturbations including antimicrobial molecules or peptides, high temperature and pH, change in membrane composition and overexpression of misfolded envelope proteins (Dorel et al., 2006; Price and Raivio, 2009; Raivio et al., 2013). These results are consistent with other studies that show the importance of genes involved in membrane structure and function for the survival of *S.* Enteritidis in EW (Lu et al., 2003; Cogan et al., 2004; Clavijo et al., 2006; Kang et al., 2006; Gantois et al., 2008a; Raspoet et al., 2014; Huang et al., 2019). Deletion of the CpxAR encoding genes demonstrated the role of CpxAR as a key regulator of *S.* Enteritidis survival under the natural alkaline conditions of EW at 37°C (Huang et al., 2019). The induction of these CpxAR-regulated genes is consistent with an attempt by *S.* Enteritidis to combat the multiple antimicrobial factors associated with EW. The similar expression response of these “cell damage and envelope stress genes” in EWF and EWMM implies that factors such as EW antimicrobial peptides of <10 kDa, high temperature and/or alkaline pH are responsible for the observed induction of this stress response. Further work is required to clarify any involvement of EW proteins of >10 kDa in this response.

However, it should be noted that there is one major difference in the membrane stress expression responses observed, since there was no induction of the CpxAR-controlled *psp* genes in EWF, unlike in EWMM (Table 2 and Figure 3).

### Egg-White Proteins Have a Minor Impact on *S.* Enteritidis Gene Expression Following Exposure for 45 min at 45°C

#### Phage-Shock Proteins

Only 64 genes showed a differential expression with respect to exposure to EWF and EWMM for 45 min at 45°C, and the expression differences observed were relatively modest (average of 3-fold). Such genes include *pspADE* and *pspG* which showed induction in EWMM but repression in EWF (Table 2 and Figure 3), indicating that EW proteins cause their induction. The *psp* response is induced by a variety of membrane-altering stresses including phage infection, heat shock, hyperosmotic shock, ethanol treatment, inhibition of fatty acid biosynthesis, exposure to hydrophobic organic solvents or proton ionophores (Joly et al., 2010 for a review). The common factor of these stresses is a negative impact on the physical/chemical properties of the membrane and it is generally assumed that induction of the *psp* response is related to the uncoupling or depletion of the pmf (Darwin, 2005; Jovanovic et al., 2006). In the present study, EW proteins were shown to be required for the disruption of the membrane potential of *S.* Enteritidis by EW, as revealed by the measurement of cytoplasmic-membrane depolarization (Figure 4). Interestingly, high temperature (45°C) alone did not have any effect on the membrane potential. Taken together, the results show that EW proteins are required for the disruption in the membrane potential by EW and for the observed *psp* induction in EWMM following exposure for 45 min at 45°C. Induction of *psp* genes by EW was not observed by Huang et al. (2019) in their investigation of the response of *S.* Enteritidis to EW exposure for 6–24 h at 37°C. One explanation for this difference might be that the *psp* response is early and transient during EW exposure, as previously reported during heat and hyperosmotic shock (Brissette et al., 1990). Previous microarray data (Baron et al., 2017) support this hypothesis: the highest *psp* induction was measured after 7 min incubation in EWMM, followed by a decrease at 25 and 45 min. This suggests that the *psp* response is rapid, which might allow *S.* Enteritidis to preserve its pmf and its membrane integrity.

#### Putrescine Metabolism

Other genes differentially regulated in EWMM and EWF (Table 2 and Figure 3) include genes with function in putrescine transport and metabolism, which suggests that EW protein exposure leads to putrescine accumulation in *S.* Enteritidis. Putrescine, like spermidine, spermine and cadaverine, belong to the polyamine family of compounds which play a crucial role in the preservation of outer membrane function and maintaining bacterial surface structures (Shah and Swiatlo, 2008 for review). Putrescine also contributes to the regulation of the pore size of outer membrane proteins, including OmpF and OmpC, as the binding of putrescine causes closure and a consequential decrease of outer membrane permeability (Iyer et al., 2000). Moreover, a *S.* Enteritidis mutant defective in secreting cadaverine, exhibits raised sensitivity to EW (Clavijo et al., 2006) indicating that cadaverine has a stabilizing effect on the cell-surface LPS, likely due to its positive charge. Likewise, the production of putrescine (positively charged at pH 9.3 due to its pKa of 10.8) may stabilize the membrane during exposure to EW proteins and this response may represent part of the global envelope stress response to EW. It should be noted that PspG-overexpression induces the up-regulation of genes involved in arginine (a putrescine precursor) biosynthesis (Jovanovic et al., 2006) and that arginine biosynthesis genes were also induced by EW proteins (Table 2). This suggests that the observed induction of genes for putrescine synthesis by EW proteins is a consequence of up-regulation of the *psp* genes in the early stage of *S.* Enteritidis response.

#### NAD Production

The up-regulation of the *nadAB* and *pnuC* genes involved in NAD production in EWMM in comparison with their expression in EWF (Table 2) may be linked to a need for NAD following exposure for 45 min at 45°C. This induction may reflect a metabolic adjustment required to maintain the NAD/NADH pool at a sufficient level since genes involved in NAD synthesis are regulated in response to NAD levels (Holley et al., 1985).

#### Phosphate Utilization

Differential expression in response to EW proteins was also observed for genes belonging to the PhoBR regulon (Table 2); such genes were significantly more down-regulated in EWF than in EWMM after 45 min at 45°C and thus their expression was enhanced by EW proteins. The overall down-regulation in the EW medium is not surprising given the high phosphate concentration of EW (5.7 mM; derived from Nys and Sauveur, 2004), much higher than that required to activate the Pho regulon (4 μM; Marzan and Shimizu, 2011). However, phosphate concentration is unlikely to explain the differential gene expression between EWF and EWMM. This suggests that exposure to EW proteins results in an increased requirement for phosphate although the precise cause of this raised demand is unclear. Nevertheless, the *phoBR* genes were down-regulated in the two media (Table 2). Interestingly, the *ugpB* gene encoding a glycerol-3-phosphate (G3P) uptake component (also belonging to the PhoBR regulon) also exhibited raised expression in EWMM with respect to EWF (this gene is up-regulated in EWMM and down-regulated in EWF, Table 2). Psp proteins have been shown to increase the pool of G3P available for phospholipid biosynthesis and as a potential source of precursor for replacement of damaged phospholipids arising from membrane stress (Jovanovic et al., 2006). This may explain the purpose of the raised *ugpB* expression as induced by EW proteins.

#### Cysteine Synthesis/Sulfur Assimilation

Eight genes (*cysDJNPUW*, *hslJ*, *tcyJ*) involved in cysteine/sulfur metabolism were significantly more down-regulated in EWF than in EWMM following exposure for 45 min at 45°C (Table 2). Several studies have shown that the *cys* regulon can take part in cellular activities which are not directly related to the biosynthesis of cysteine and assimilation of sulfur, such as resistance to antibiotics (Lilic et al., 2003 for review). The *hslJ* gene, belonging to the *cys* regulon, has been shown to be involved in novobiocin resistance and the *psp* response in *E. coli* (Lilic et al., 2003). The “mislocation” of HslJ, induced by a membrane stress, leads to an increased *psp* response (Joly et al., 2010). Further studies will be necessary to assess whether the differential expression observed here for cysteine/sulfur-metabolism-related genes is linked to the *psp* response. It is also possible that these results are related to raised sulfur and cysteine availability due to the presence of EW proteins.

#### Zinc Uptake

EWMM induced the expression of the *znuABC* and *zinT* genes after 45 min at 45°C. These genes are repressed in response to zinc by the transcriptional repressor Zur (Gabbianelli et al., 2011). Zinc homeostasis is essential as many proteins, such as DNA and RNA polymerases, ribosomal proteins and multiple metabolic enzymes, are strictly Zn dependent. The transcription of the *znuA* gene is induced when bacteria are cultivated in Zn concentrations below 0.5 μM (Ammendola et al., 2007), which is a much lower level than that reported in EW (18 μM derived from Nys and Sauveur, 2004). This suggests that the availability of Zn in EWMM is lower than that in EWF. Ovotransferrin is known to bind divalent cations and has Zn-sequestering activity (Tan and Woodworth, 1969), and so could compete for Zn with *S*. Enteritidis in EWMM which would provide an explanation for the apparent reduced Zn availability in EWMM vs. EWF. However, Zn deficiency is expected to be the same in EWMM and in EWF since Zn bound to ovotransferrin in EW should be removed with ovotransferrin in the ultrafiltration step; thus, the cause of repression for the Zn-uptake related genes in EWF remains unclear.

#### Lysozyme Responsive Genes

The gene encoding the lysozyme inhibitor protein was induced by the presence of EW proteins (containing lysozyme). The lysozyme inhibitor protein protects Gram-negative bacteria against lysozyme when the outer membrane is permeated (Deckers et al., 2004; Callewaert et al., 2008). A lysozyme inhibitor knock-out mutant exhibited raised susceptibility to the antimicrobial activity of lysozyme (Callewaert et al., 2008), which was further raised by the additional presence of lactoferrin, a transferrin/ovotransferrin-related protein able to permeabilize the outer membrane through its cation-chelating activity. Moreover, Wang et al. (2018) showed that *S.* Enteritidis survival in EW is reduced by deletion of the lysozyme inhibitor gene. Therefore, it can be hypothesized that 45 min exposure to EWMM at 45°C led to permeabilization of the outer membrane of *S.* Enteritidis (probably through the action of ovotransferrin, which is known to chelate divalent cations) which in turn enabled lysozyme to gain access the peptidoglycan stimulating the induction of lysozyme inhibitor gene in response.

### The Role of EW Proteins in the Antibacterial Activity of EW at 45°C

#### Membrane Depolarization by EW Proteins

Among the genes exhibiting differential expression at the early stage of *S.* Enteritidis incubation in the two media at 45°C, the *psp* genes are known to be induced by change in membrane status. Moreover, the differential induction of genes for putrescine synthesis, phosphate utilization and cysteine/sulfur metabolism, are probably a consequence of up-regulation of the *psp* genes. In addition, the regulation of the lysozyme-inhibitor response genes after 45 min at 45°C is also suggestive of membrane perturbation. Consequently, membrane depolarization experiments were performed to investigate perturbation of the membrane pmf, as the EW-protein-induced effect on the membrane was the most prominent observed. The results provided here further show that EW proteins are required for the disruption of the electrochemical potential of the inner membrane by EW. Among EW proteins, ovotransferrin may be the cause of these changes. As indicated above, divalent cations within the outer membrane are critical for stabilization of the outer membrane through neutralization of the highly negatively charged lipopolysaccharides. By chelating these membrane-associated cations, metal-binding proteins such as ovotransferrin impair outer membrane integrity (Ellison et al., 1988). In addition, transferrins can permeate the outer membrane of *E. coli* and access the inner membrane causing permeation of ions, leading to a decrease in membrane electrochemical potential (Aguilera et al., 2003). More recently, ovotransferrin was shown to provoke a perturbation of the membrane electrochemical potential and membrane dysfunction in the Gram-positive bacterium *Bacillus cereus* under the natural alkaline pH conditions of EW (Baron et al., 2014). EW may also contain other proteins able to permeabilize and disrupt the membrane. For example, three proteins belonging to Bactericidal Permeability Increasing protein/Lipopolysaccharide Binding Protein (BPI/LBP) family have been identified in hen EW (Baron et al., 2016 for review) including the protein Tenp (Transiently Expressed in Neural Precursor); this protein is estimated to represent 0.1–0.5% of total EW proteins (Guérin-Dubiard et al., 2006). Further studies are required to clarify the role of ovotransferrin, alone or in combination with other EW molecules, in the observed membrane disruption activity of EW proteins, as well as the *psp* response, during EW incubation at 45°C.

However, despite the membrane depolarization effect of EW proteins observed, this appears not to be the only mechanism of cell death since cell death still occurred in EWF (depleted in proteins of > 10 kDa) where no membrane depolarization was observed. It is important to note that the fluorescent test using DiSC_3_(5) measures the membrane depolarization and not membrane destruction or cell death *per se*. This view is supported by the observation that valinomycin can dissipate the membrane potential (according to DiSC3(5) assays) but is not lethal at the concentrations utilized (1 μM) (Wu et al., 1999).

#### The *S*. Enteritidis Killing Activity of Egg White at 45°C

Since no notable *S.* Enteritidis cell death occurred in TSB at 45°C, the drastic decline in survival in EWF suggests that the ionic environment of EWF, and/or one or more of the small (< 10 kDa) bioactive proteins (or polypeptides) of EW, are involved in the bactericidal activity observed at 45°C in EWF, EWMM and EW. Of the many proteins identified in EW, only the ∼7 kDa ß-defensins polypeptides are sufficiently small to be able to pass through a 10 kDa cut-off membrane. To date, two avian ß-defensins have been found in EW: AvBD11 and gallin (Man, 2007). AvBD11 and Gallin inhibit the growth of both *S.* Enteritidis in TSA (Hervé-Grépinet et al., 2010) and *E. coli* in phosphate buffered saline (PBS) at pH 7 (Gong et al., 2010). However, their activity under the alkaline pH and ionic environment of EW has yet to be explored nor has their contribution to the bactericidal activity of EWF toward *S.* Enteritidis at 45°C. Moreover, it is possible that EW contains unknown antimicrobial polypeptides of low mass (<10 kDa) which could also play a part in the bactericidal activity of EW at 45°C. Studies on the survival of an *S.* Enteritidis *yoaE* mutant found that proteinase K treatment eradicated the antibacterial activity of a 3-kDa EW filtrate (Huang et al., 2020). This result suggests that antimicrobial polypeptides of <3 kDa (and therefore < 10 kDa) play an active role in the antibacterial defense of EW, highlighting the need for further investigation.

## Conclusion

The present study was conducted to better understand the mechanism of *S.* Enteritidis killing in EW at 45°C and to explore the impact of EW proteins. In contrast to many other such studies, the conditions applied here were chosen to mimic the highly efficient process patented by Liot and Anza (1996) for liquid EW stabilization. Surprisingly, EWF (EW protein-depleted) exhibited a bactericidal activity at 45°C similar to that of EW indicating that EW proteins play a minor role in the bactericidal activity of EW at 45 C. However, EW proteins were essential for the disruption of the membrane potential and to the induction of the *psp* response at the early stage of incubation at 45°C (although only a modest overall expression response to EW proteins was observed). Further research is required to determine the key factors of EWF involved in its bactericidal activity at 45°C, with the ionic environment of EWF and its antimicrobial peptides being obvious candidates. The research presented here underlines the complex, multifactorial and highly effective bactericidal mechanisms of EW and highlights our lack of understanding of the key factors involved. *S.* Enteritidis remains a major threat in egg-related salmonellosis and there is a need to better control this foodborne pathogen. A more thorough comprehension of the antibacterial mechanisms of EW should assist this requirement.

## Data Availability Statement

The datasets generated for this study can be found in the online repositories. The names of the repository/repositories and accession number(s) can be found below: https://www.ncbi.nlm.nih.gov/geo/query/acc.cgi?acc=GSE144179.

## Author Contributions

M-FC: planning and carrying out survival, qRT-PCR and membrane depolarization experiments, and drafting a part of the “Materials and Methods” section. MA: planning and carrying out experiments, analysis, and interpretation of the microarray data. FB, SB, SJ, MG, FN, CG-D, and SA: conception and design of the work, design of experimentation, data interpretation, and drafting a part of “Results” or “Discussion” section (according to the specific speciality of each author: microbiology, molecular biology, biochemistry, iron metabolism). FB, SB, and SA have revised the entire manuscript critically for important intellectual content. All authors contributed to the article and approved the submitted version.

## Conflict of Interest

The authors declare that the research was conducted in the absence of any commercial or financial relationships that could be construed as a potential conflict of interest.

## Footnotes

1 http://cran.r-project.org/

2 http://www.ncbi.nlm.nih.gov/nuccore/AM933172.1

3 https://earray.chem.agilent.com/earray

4 https://www.ncbi.nlm.nih.gov/geo/query/acc.cgi?acc=GSE144179

5 http://www.ncbi.nlm.nih.gov/geo/query/acc.cgi?acc=GSE92545

6 http://www.r-project.org/

7 http://www.ncbi.nlm.nih.gov/COG

8 http://primer3.ut.ee/

9 https://genorm.cmgg.be

## References

[B1] Aguilera O. Quiros L. M. Fierro J. F. (2003). Transferrins selectively cause ion efflux through bacterial and artificial membranes. *FEBS Lett.* 548 5–10. 10.1016/S0014-5793(03)00719-1 12885398

[B2] Alabdeh M. Lechevalier V. Nau F. Gautier M. Cochet M.-F. Gonnet F. (2011). Role of incubation conditions and protein fraction on the antimicrobial activity of egg white against *Salmonella* Enteritidis and *Escherichia coli*. *J. Food Prot.* 74 24–31. 10.4315/0362-028x.jfp-10-157 21219759

[B3] Ammendola S. Pasquali P. Pistoia C. Petrucci P. Petrarca P. Rotilio G. (2007). High-affinity Zn^2+^ Uptake System ZnuABC is required for bacterial Zinc homeostasis in intracellular environments and contributes to the virulence of *Salmonella enterica*. *Infect. Immun.* 75 5867–5876. 10.1128/IAI.00559-07 17923515 PMC2168356

[B4] Andrews S. C. Robinson A. K. Rodriguez-Quinones F. (2003). Bacterial iron homeostasis. *Fems Microbiol. Rev.* 27 215–237. 10.1016/s0168-6445(03)00055-x 12829269

[B5] Banks J. G. Board R. G. Sparks N. H. (1986). Natural antimicrobial systems and their potential in food preservation of the future. *Biotechnol. Appl. Biochem.* 8 103–147. 3017380

[B6] Baron F. (2010). “Qualité microbiologique de l’oeuf,” in *Science et technologie de l’oeuf*? ed. Lavoisier A (Paris: Lavoisier), 315–350

[B7] Baron F. Bonnassie S. Alabdeh M. Cochet M.-F. Nau F. Guérin-Dubiard C. (2017). Global gene-expression analysis of the response of *Salmonella* Enteritidis to egg-white exposure reveals multiple egg-white-imposed stress responses. *Front. Microbiol.* 8:829. 10.3389/fmicb.2017.00829 28553268 PMC5428311

[B8] Baron F. Cochet M.-F. Ablain W. Grosset N. Madec M. N. Gonnet F. (2006). Rapid and cost-effective method for microorganism enumeration based on miniaturization of the conventional plate-counting technique. *Lait* 86 251–257. 10.1051/lait:2006005

[B9] Baron F. Gautier M. Brule G. (1997). Factors involved in the inhibition of growth of *Salmonella* Enteritidis in liquid egg white. *J. Food Prot.* 60 1318–1323. 31207765 10.4315/0362-028X-60.11.1318

[B10] Baron F. Jan S. Gonnet F. Pasco M. Jardin J. Giudici B. (2014). Ovotransferrin plays a major role in the strong bactericidal effect of egg white against the *Bacillus cereus* group. *J. Food Prot.* 77 955–962. 10.4315/0362-028X.JFP-13-473 24853518

[B11] Baron F. Nau F. Guérin-Dubiard C. Bonnassie S. Gautier M. Andrews S. C. (2016). Egg white versus *Salmonella* Enteritidis?! A harsh medium meets a resilient pathogen. *Food Microbiol.* 53(Pt B) 82–93. 10.1016/j.fm.2015.09.009 26678134

[B12] Bjarnason J. Southward C. M. Surette M. G. (2003). Genomic profiling of iron-responsive genes in *Salmonella enterica* serovar Typhimurium by high-throughput screening of a random promoter library. *J. Bacteriol.* 185 4973–4982. 10.1128/jb.185.16.4973-4982.2003 12897017 PMC166456

[B13] Brissette J. L. Russel M. Weiner L. Model P. (1990). Phage shock protein, a stress protein of *Escherichia coli*. *Proc. Natl. Acad. Sci. U.S.A.* 87 862–866. 10.1073/pnas.87.3.862 2105503 PMC53368

[B14] Callewaert L. Aertsen A. Deckers D. Vanoirbeek K. G. A. Vanderkelen L. Van Herreweghe J. M. (2008). A new family of lysozyme inhibitors contributing to lysozyme tolerance in gram-negative bacteria. *PLoS Pathog.* 4:e1000019. 10.1371/journal.ppat.1000019 18369469 PMC2267010

[B15] Chen J. Thesmar H. S. Kerr W. L. (2005). Outgrowth of *Salmonellae* and the physical property of albumen and vitelline membrane as influenced by egg storage conditions. *J. Food Prot.* 68 2553–2558. 10.4315/0362-028x-68.12.2553 16355825

[B16] Clavijo R. I. Loui C. Andersen G. L. Riley L. W. Lu S. (2006). Identification of genes associated with survival of *Salmonella enterica* serovar Enteritidis in chicken egg albumen. *Appl. Environ. Microbiol.* 72 1055–1064. 10.1128/AEM.72.2.1055-1064.2006 16461649 PMC1392908

[B17] Clay C. E. Board R. G. (1991). Growth of *Salmonella* Enteritidis in artificially contaminated hens’ shell eggs. *Epidemiol. Infect.* 106 271–281. 10.1017/S095026880004841X 2019298 PMC2271999

[B18] Cogan T. A. Domingue G. Lappin-Scott H. M. Benson C. E. Woodward M. J. Humphrey T. J. (2001). Growth of *Salmonella* Enteritidis in artificially contaminated eggs: the effects of inoculum size and suspending media. *Int. J. Food Microbiol.* 70 131–141. 10.1016/S0168-1605(01)00540-2 11759751

[B19] Cogan T. A. Jørgensen F. Lappin-Scott H. M. Benson C. E. Woodward M. J. Humphrey T. J. (2004). Flagella and curli fimbriae are important for the growth of *Salmonella enterica* serovars in hen eggs. *Microbiology* 150 1063–1071. 10.1099/mic.0.26791-0 15073315

[B20] Darwin A. J. (2005). The phage-shock-protein response: the Psp response. *Mol. Microbiol.* 57 621–628. 10.1111/j.1365-2958.2005.04694.x 16045608

[B21] De Vylder J. Raspoet R. Dewulf J. Haesebrouck F. Ducatelle R. Van Immerseel F. (2013). *Salmonella* Enteritidis is superior in egg white survival compared with other *Salmonella* serotypes. *Poult. Sci. Assoc. Inc.* 92 842–845. 10.3382/ps.2012-02668 23436537

[B22] Deckers D. Masschalck B. Aertsen A. Callewaert L. Van Tiggelen C. G. M. (2004). Periplasmic lysozyme inhibitor contributes to lysozyme resistance in *Escherichia coli*. *Cell. Mol. Life Sci.* 61 1229–1237. 10.1007/s00018-004-4066-3 15141308 PMC11138759

[B23] Delcour A. H. (2009). Outer membrane permeability and antibiotic resistance. *Biochim. Biophys. Acta BBA Proteins Proteomics* 1794 808–816. 10.1016/j.bbapap.2008.11.005 19100346 PMC2696358

[B24] Dorel C. Lejeune P. Rodrigue A. (2006). The Cpx system of *Escherichia coli*, a strategic signaling pathway for confronting adverse conditions and for settling biofilm communities? *Res. Microbiol.* 157 306–314. 10.1016/j.resmic.2005.12.003 16487683

[B25] EFSA Biohaz Panel (European Food Safety Autority Panel on Biological Hazards), (2014). Scientific Opinion on the public health risks of table eggs due to deterioration and development of pathogens. *EFSA J.* 12:3782. 10.2903/j.efsa.2014.3782 29606757

[B26] Ellison R. T. Giehl T. J. LaForce F. M. (1988). Damage of the outer membrane of enteric Gram-negative bacteria by lactoferrin and transferrin. *Infect. Immun.* 56 2774–2781. 3169987 10.1128/iai.56.11.2774-2781.1988 PMC259649

[B27] Epand R. F. Pollard J. E. Wright J. O. Savage P. B. Epand R. M. (2010). Depolarization, bacterial membrane composition and the antimicrobial action of ceragenins. *Antimicrob. Agents Chem.* 54 3708–3713. 10.1128/AAC.00380-10 20585129 PMC2934994

[B28] Franchini A. G. (2006). Global gene expression in *Escherichia coli* K-12 during short-term and long-term adaptation to glucose-limited continuous culture conditions. *Microbiology* 152 2111–2127. 10.1099/mic.0.28939-0 16804185

[B29] Gabbianelli R. Scotti R. Ammendola S. Petrarca P. Nicolini L. Battistoni A. (2011). Role of ZnuABC and ZinT in *Escherichia coli O157*:H7 zinc acquisition and interaction with epithelial cells. *BMC Microbiol.* 11:36. 10.1186/1471-2180-11-36 21338480 PMC3053223

[B30] Gantois I. Ducatelle R. Pasmans F. Haesebrouck F. Gast R. Humphrey T. J. (2009). Mechanisms of egg contamination by *Salmonella* Enteritidis. *FEMS Microbiol. Rev.* 33 718–738. 10.1111/j.1574-6976.2008.00161.x 19207743

[B31] Gantois I. Ducatelle R. Pasmans F. Haesebrouck F. Immerseel F. V. (2008a). *Salmonella enterica* serovar Enteritidis genes induced during oviduct colonization and egg contamination in laying hens. *Appl. Environ. Microbiol.* 74 6616–6622. 10.1128/AEM.01087-08 18776023 PMC2576714

[B32] Gantois I. Eeckhaut V. Pasmans F. Haesebrouck F. Ducatelle R. Immerseel F. V. (2008b). A comparative study on the pathogenesis of egg contamination by different serotypes of *Salmonella*. *Avian Pathol.* 37 399–406. 10.1080/03079450802216611 18622856

[B33] Garibaldi J. A. (1970). Role of microbial iron transport compounds in bacterial spoilage of eggs. *Appl. Microbiol.* 20 558–560. 5498603 10.1128/am.20.4.558-560.1970 PMC376988

[B34] Gong D. Wilson P. W. Bain M. M. McDade K. Kalina J. Herve-Grépinet V. (2010). Gallin; an antimicrobial peptide member of a new avian defensin family, the ovodefensins, has been subject to recent gene duplication. *BMC Immunol*. 11. 10.1186/1471-2172-11-12 20226050 PMC2846878

[B35] Guan J. Grenier C. Brooks B. W. (2006). In vitro study of *Salmonella* Enteritidis and *Salmonella* Typhimurium definitive type 104: survival in egg albumen and penetration through the vitelline membrane. *Poult. Sci.* 85 1678–1681. 16977857 10.1093/ps/85.9.1678

[B36] Guérin-Dubiard C. Pasco M. Mollé D. Désert C. Croguennec T. Nau F. (2006). Proteomic analysis of hen egg white. *J. Agric. Food Chem.* 54 3901–3910. 10.1021/jf0529969 16719513

[B37] Hervé-Grépinet V. Réhault-Godbert S. Labas V. Magallon T. Derache C. Lavergne M. (2010). Purification and characterization of avian beta-defensin 11, an antimicrobial peptide of the hen egg. *Antimicrob. Agents Chemother.* 54 4401–4409. 10.1128/AAC.00204-10 20625158 PMC2944589

[B38] Holley E. A. Spector M. P. Foster J. W. (1985). Regulation of NAD biosynthesis in *Salmonella* Typhimurium: expression of nad-lac gene fusions and identification of a nad regulatory locus. *J. Gen. Microbiol.* 131 2759–2770. 3934331 10.1099/00221287-131-10-2759

[B39] Huang X. Hu M. Zhou X. Liu Y. Shi C. Shi X. (2020). Role of *yoaE* gene regulated by CpxR in the survival of *Salmonella enterica* Serovar Enteritidis in antibacterial egg white. *mSphere* 5:e00638-19. 10.1128/mSphere.00638-19 31915212 PMC6952189

[B40] Huang X. Zhou X. Jia B. Li N. Jia J. He M. (2019). Transcriptional sequencing uncovers survival mechanisms of *Salmonella enterica* Serovar Enteritidis in antibacterial egg white. *mSphere* 4 700–718. 10.1128/mSphere.00700-18 30760616 PMC6374596

[B41] Humphrey T. J. Whitehead A. (1993). Egg age and the growth of *Salmonella* Enteritidis PT4 in egg contents. *Epidemiol. Infect.* 111 209–219. 8405149 10.1017/s0950268800056910 PMC2271372

[B42] Iyer R. Wu Z. Woster P. M. Delcour A. H. (2000). Molecular basis for the polyamine-OmpF porin interactions: inhibitor and mutant studies. *J. Mol. Biol.* 297 933–945. 10.1006/jmbi.2000.3599 10736228

[B43] Jan S. Baron F. Alabdeh M. Chaari W. Grosset N. Cochet M.-F. (2013). Biochemical and micrographic evidence of *Escherichia coli* membrane damage during incubation in egg white under bactericidal conditions. *J. Food Prot.* 76 1523–1529. 10.4315/0362-028X.JFP-12-418 23992496

[B44] Joly N. Engl C. Jovanovic G. Huvet M. Toni T. Sheng X. (2010). Managing membrane stress: the phage shock protein (Psp) response, from molecular mechanisms to physiology. *FEMS Microbiol. Rev.* 34 797–827. 10.1111/j.1574-6976.2010.00240.x 20636484

[B45] Jovanovic G. Lloyd L. J. Stumpf M. P. H. Mayhew A. J. Buck M. (2006). Induction and function of the phage shock protein extracytoplasmic stress response in *Escherichia coli*. *J. Biol. Chem.* 281 21147–21161. 10.1074/jbc.M602323200 16709570

[B46] Kang H. Loui C. Clavijo R. I. Riley L. W. Lu S. (2006). Survival characteristics of *Salmonella enterica* serovar Enteritidis in chicken egg albumen. *Epidemiol. Infect.* 134:967. 10.1017/S0950268806006054 16650332 PMC2870490

[B47] Lilic M. Jovanovic M. Jovanovic G. Savic D. J. (2003). Identification of the CysB−regulated gene, *hslJ*, related to the *Escherichia coli* novobiocin resistance phenotype. *FEMS Microbiol. Lett.* 224 239–246. 10.1016/S0378-1097(03)00441-5 12892888

[B48] Liot R. Anza L. (1996). *Procédé de Traitement de Blanc d’oeuf Liquide*. French Patent no 9608356. Paris.

[B49] Lock J. L. Board R. G. (1992). Persistence of contamination of hens’ egg albumen in vitro with *Salmonella* serotypes. *Epidemiol. Infect.* 108 389–396. 1601073 10.1017/s095026880004989x PMC2272201

[B50] Lu S. W. Killoran P. B. Riley L. W. (2003). Association of *Salmonella enterica* serovar Enteritidis YafD with resistance to chicken egg albumen. *Infect. Immun.* 71 6734–6741. 10.1128/iai.71.12.6734-6741.2003 14638758 PMC308936

[B51] Man K. (2007). The chicken egg white proteome. *Proteomics* 7 3558–3568. 10.1002/pmic.200700397 17722208

[B52] Marzan L. W. Shimizu K. (2011). Metabolic regulation of *Escherichia coli* and its phoB and phoR genes knockout mutants under phosphate and nitrogen limitations as well as at acidic condition. *Microbial Cell Factories* 10:39. 10.1186/1475-2859-10-39 21599905 PMC3129296

[B53] McHugh J. P. Rodriguez-Quinones F. Abdul-Tehrani H. Svistunenko D. A. Poole R. K. Cooper C. E. (2003). Global iron-dependent gene regulation in *Escherichia coli*–a new mechanism for iron homeostasis. *J. Biol. Chem.* 278 29478–29486. 10.1074/jbc.M303381200 12746439

[B54] Murase T. Holt P. S. Gast R. K. (2005). Growth of *Salmonella enterica* serovar Enteritidis in albumen and yolk contents of eggs inoculated with this organism onto the vitelline membrane. *J. Food Prot.* 68 718–721. 15830661 10.4315/0362-028x-68.4.718

[B55] Nys Y. Sauveur B. (2004). *Valeur nutritionnelle des oeufs.* Available online at: http://prodinra.inra.fr/?locale=fr#!ConsultNotice:77361 (accessed February 17, 2016).

[B56] Price N. L. Raivio T. L. (2009). Characterization of the Cpx regulon in *Escherichia coli* strain MC4100. *J. Bacteriol.* 191 1798–1815. 10.1128/JB.00798-08 19103922 PMC2648356

[B57] Qin X. He S. Zhou X. Cheng X. Huang X. Wang Y. (2019). Quantitative proteomics reveals the crucial role of YbgC for *Salmonella enterica* serovar Enteritidis survival in EW. *Int. J. Food. Microbiol.* 289 115–126. 10.1016/j.ijfoodmicro.2018.08.010 30223195

[B58] Raivio T. L. Leblanc S. K. D. Price N. L. (2013). The *Escherichia coli* Cpx envelope stress response regulates genes of diverse function that impact antibiotic resistance and membrane integrity. *J. Bacteriol.* 195 2755–2767. 10.1128/JB.00105-13 23564175 PMC3697260

[B59] Raspoet R. Appia-Ayme C. Shearer N. Martel A. Pasmans F. Haesebrouck F. (2014). Microarray-based detection of *Salmonella enterica* serovar Enteritidis genes involved in chicken reproductive tract colonization. *Appl. Environ. Microbiol.* 80 7710–7716. 10.1128/AEM.02867-14 25281378 PMC4249237

[B60] Rodionov D. A. Li X. Rodionova I. A. Yang C. Sorci L. Dervyn E. (2008). Transcriptional regulation of NAD metabolism in bacteria: genomic reconstruction of NiaR (YrxA) regulon. *Nucleic Acids Res.* 36 2032–2046. 10.1093/nar/gkn046 18276644 PMC2330245

[B61] Růzicková V. (1994). Growth and survival of *Salmonella* Enteritidis in selected egg foods. *Veterinární Med.* 39 187–195. 8085304

[B62] Schneider J. Wendisch V. F. (2011). Biotechnological production of polyamines by bacteria: recent achievements and future perspectives. *Appl. Microbiol. Biotechnol.* 91 17–30. 10.1007/s00253-011-3252-0 21552989

[B63] Schoeni J. L. Glass K. A. McDermott J. L. Wong A. C. (1995). Growth and penetration of *Salmonella enteritidis*, *Salmonella heidelberg* and *Salmonella typhimurium* in eggs. *Int. J. Food Microbiol.* 24, 385–396. 10.1016/0168-1605(94)00042-5 7710915

[B64] Shah P. Swiatlo E. (2008). A multifaceted role for polyamines in bacterial pathogens. *Mol. Microbiol.* 68 4–16. 10.1111/j.1365-2958.2008.06126.x 18405343

[B65] Smyth G. K. (2004). Linear models and empirical bayes methods for assessing differential expression in microarray experiments. *Stat. Appl. Genet. Mol. Biol*. 3 1–25. 10.2202/1544-6115.1027 16646809

[B66] Tan A. T. Woodworth R. C. (1969). Ultraviolet difference spectral studies of conalbumin complexes with transition metal ions. *Biochemistry* 8 3711–3716. 5387528 10.1021/bi00837a033

[B67] Wang Y. Jia B. Xu X. Zhang L. Wei C. Ou H. (2018). Comparative genomic analysis and characterization of two *Salmonella enterica* Serovar Enteritidis isolates from poultry with notably different survival abilities in egg whites. *Front. Microbiol.* 9:2111. 10.3389/fmicb.2018.02111 30245675 PMC6137255

[B68] Wu M. Maier E. Benz R. Hancock R. E. W. (1999). Mechanism of interaction of different classes of cationic antimicrobial peptides with planar bilayers and with the cytoplasmic membrane of *Escherichia coli*. *Biochemistry* 38 7235–7242. 10353835 10.1021/bi9826299

